# Risk factors and predictors of lymph nodes metastasis and distant metastasis in newly diagnosed T1 colorectal cancer

**DOI:** 10.1002/cam4.3114

**Published:** 2020-05-29

**Authors:** Kaibo Guo, Yuqian Feng, Li Yuan, Harpreet S. Wasan, Leitao Sun, Minhe Shen, Shanming Ruan

**Affiliations:** ^1^ The First Clinical Medical College of Zhejiang Chinese Medical University Hangzhou Zhejiang P.R. China; ^2^ Department of Cancer Medicine Hammersmith Hospital Imperial College Healthcare NHS Trust London UK; ^3^ Department of Medical Oncology The First Affiliated Hospital of Zhejiang Chinese Medical University Hangzhou Zhejiang P.R. China

**Keywords:** distant metastasis, lymph nodes metastasis, nomogram, T1 colorectal cancer

## Abstract

**Background:**

Lymph nodes metastasis (LNM) and distant metastasis (DM) are important prognostic factors in colorectal cancer (CRC) and determine the following treatment approaches. We aimed to find clinicopathological factors associated with LNM and DM, and analyze the prognosis of CRC patients with T1 stage.

**Methods:**

A total of 17 516 eligible patients with T1 CRC were retrospectively enrolled in the study based on the Surveillance, Epidemiology, and End Results (SEER) database during 2004‐2016. Logistic regression analysis was performed to identify risk factors for LNM and DM. Unadjusted and adjusted Cox proportional hazard models were used to identify prognostic factors for overall survival. We performed the cumulative incidence function (CIF) to further determine the prognostic role of LNM and DM in colorectal cancer‐specific death (CCSD). LNM, DM, and OS nomogram were constructed based on these models and evaluated by the C‐index and calibration plots for discrimination and accuracy, respectively. The clinical utility of the nomograms was measured by decision curve analyses (DCAs) and subgroups with different risk scores.

**Results:**

Tumor grade, mucinous adenocarcinoma, and age accounted for the first three largest proportion among the LNM nomogram scores (all, *P* < .001), whereas N stage, carcinoembryonic antigen (CEA), and tumor size occupied the largest percentage in DM nomogram (all, *P* < .001). OS nomogram was formulated to visually to predict 3‐, 5‐, and 10‐ year overall survivals for patients with T1 CRC. The calibration curves showed an effectively predictive accuracy of prediction nomograms, of which the C‐index were 0.666, 0.874, and 0.760 for good discrimination, respectively. DCAs and risk subgroups revealed the clinical effectiveness of these nomograms.

**Conclusions:**

Novel population‐based nomograms for T1 CRC patients could objectively and accurately predict the risk of LNM and DM, as well as OS for different stages. These predictive tools may help clinicians to make individual clinical decisions, before clinical management.

## Graphical abstract

In this study, we analyzed 17 516 participants collected from the Surveillance, Epidemiology, and End Results (SEER) database. Then, we construct the nomograms to predict the probability of lymph nodes metastasis in colorectal cancer with T1N0‐2M0 stage, and to estimate the probability of distant metastasis in T1 colorectal cancer.

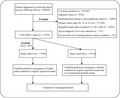

## 1 INTRODUCTION

Colorectal cancer (CRC) accounts for approximately 10% of all annually diagnosed cancers and cancer‐related deaths in the world.^1^ In 2019, colorectal cancer is estimated to rank the third highest cancer incidence and mortality in USA.^2^ Based on the 8th American Joint Committee on Cancer (AJCC) TNM staging system, T1 colorectal carcinoma refers that primary tumor only invades submucosa. Interestingly, T1 colorectal carcinoma is nonhomogeneous in the clinical features and prognosis. A small number of T1 colorectal cancer patients (CRCs) occur lymph nodes metastasis (LNM) or distant metastasis (DM), which are significantly associated with the poor prognosis. Thus, the probability of lymph nodes involvement and distant metastasis needs to be considered in the clinical management of CRC.

Generally, endoscopic treatment, surgical resection, and systemic therapy have become the main therapeutic approaches for CRC at different stages.^3^, ^4^, ^5^ Endoscopic resection that is both safer and less expensive than surgery,^6^ is a viable option for T1 CRCs. After initial endoscopic resection, approximately 10% T1 CRCs are at III or IV stage^7^, ^8^, ^9^ and we must try to exclude these risk factors from T1N0M0 to determine if subsequent treatment is needed. For T1N1‐2M0 CRCs, endoscopic treatment could not solve the problem of positive regional lymph nodes and surgery is the cornerstone of curative intent treatment for CRCs with III stage.^10^ Systemic treatment including chemotherapies, targeted therapies, and immunotherapy, might benefit T1 CRCs with distant metastasis, regardless of the pathologic N classification.^11^

Based on the heterogeneity of T1 CRCs, as well as completely different treatment, it is necessary to construct predictive models of LNM, DM, and OS, and to establish an appropriate therapeutic strategy for CRCs. Therefore, we aimed to evaluate T1 CRCs by LNM, DM, and OS nomograms developed in the Surveillance, Epidemiology, and End Results (SEER) database.

## 2 METHODS

### 2.1 Patient enrollment and characteristics

**Figure 1:**
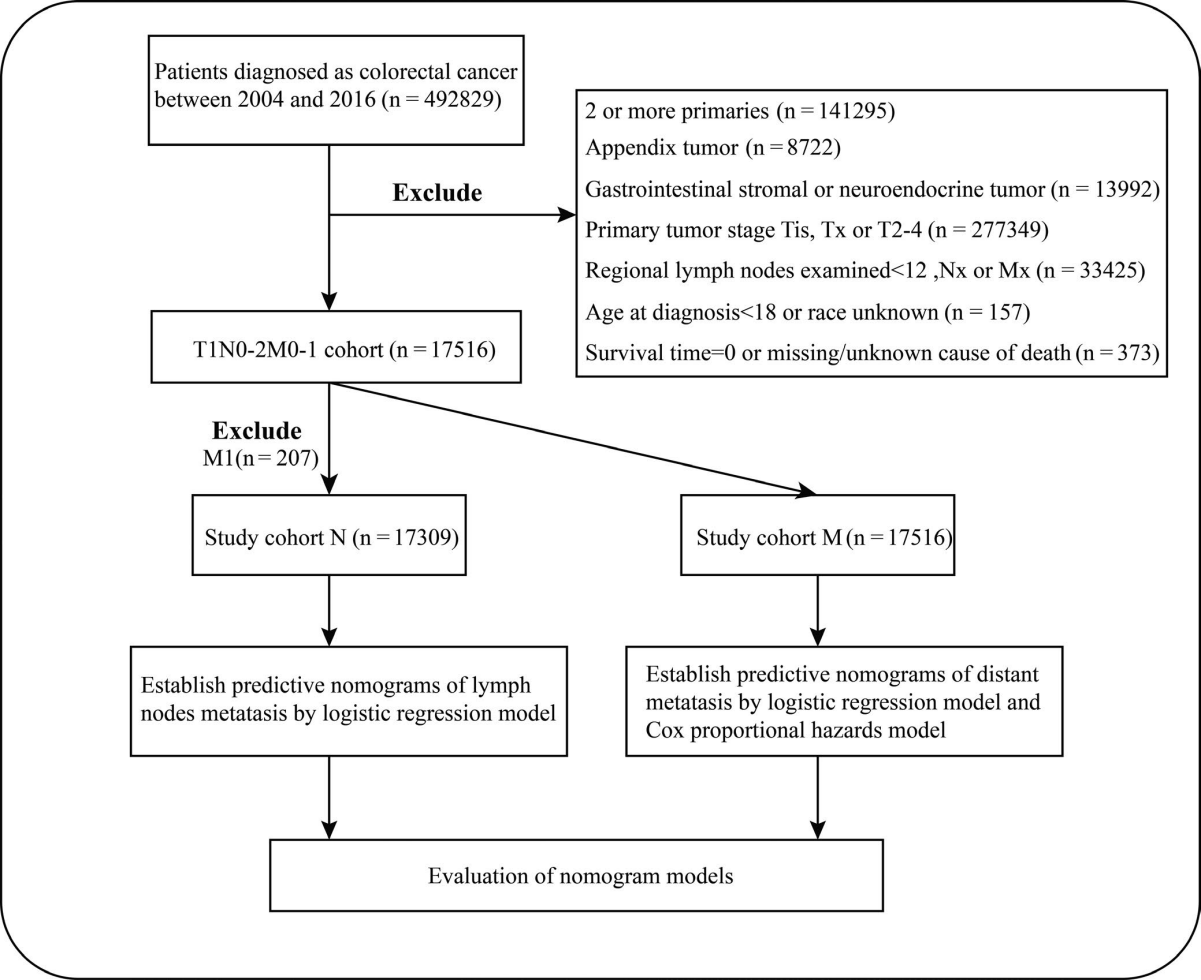
Research flowchart

The records of patients were downloaded from the SEER 18 registry database using SEER*Stat 8.3.6 software (http://seer.cancer.gov/seerstat/). SEER database currently collects and publishes cancer incidence and survival data covering approximately 34.6 percent of the US population (https://seer.cancer.gov/about/overview.html). Within the SEER database, we identified 17 516 adult patients who were diagnosed as suffering from only one primary, T1 colorectal cancer from January 2004 to December 2016. The flowchart of cases selection is shown in Figure 1. Colorectal carcinoma cases were screened by International Classification of Diseases for Oncology, 3rd Edition (ICD‐O‐3) Hist/behav, malignant. The TNM staging data were retrieved based on the American Joint Committee on Cancer (AJCC) 7th. Other characteristics at diagnosis of all patients were obtained, including year of diagnosis, age at diagnosis, race, gender, marital status, tumor location, histology, tumor size, regional nodes examined, grade, survival status, carcinoembryonic antigen (CEA), and follow‐up time. Patients with carcinoma in situ or T2‐4, unknown T, N, or M status were not included in the cohort. Cases with regional nodes examined < 12^12^ or incomplete data of survival information (time and cause of death) were also excluded. Based on existing evidence‐based medicine, LNM status is a significant prognosis factor for T1 CRC patients without M1, whereas patients with M1 (T1NXM1) could be identified as stage IV and LNM status would not determine the treatment. So we further divided the patients into two study sets, forming T1N0‐2M0 CRC population named the study cohort N (n = 17 309) for predicting LNM and T1 CRC population called the study cohort M (n = 17 516) for predicting DM. No ethical approval was sought for this study, as the data used were collected from the public SEER database, which is available as open‐access and anonymized data.

### 2.2 Variable declaration

The tumor locations were grouped into side (Cecum, ascending colon, hepatic flexure of colon), left colon (splenic flexure of colon, descending colon, sigmoid colon, rectosigmoid junction, and rectum), and unknown (not stated, transverse colon). The histology variable was classified as “Adenocarcinoma,” “Mucinous adenocarcinoma,” or “Other/Not stated.”

### 2.3 Nomogram construction and validation

Univariable and multivariable analyses were performed to identify independent risk factors and prognostic factors in T1 colorectal carcinoma in the SEER cohort. Binary logistic regression models^13^ were used to identify risk factors of LNM in the study cohort N and DM in the study cohort M, respectively. Cox proportional hazard model^14^ was conducted to identify potentially important prognostic factors for patients with T1 CRC. The Kaplan‐Meier method was used for plotting overall survival curves. Cumulative incidence function was applied for plotting cancer‐specific cumulative incidence. Based on the multivariate binary logistic regression models and Cox proportional hazard model, three novel nomograms^15^ were established and validated by the concordance index (C‐index) and calibration plots conducted by a bootstrapping method with 1000 resamples. The C‐index was used to quantify the discriminatory power of the model and the calibration plots was used to evaluate the accuracy of the nomograms. The clinical application value of the nomogram models was determined by decision curve analyses (DCAs) that calculate the net benefits at each risk threshold probability. Additionally, based on the DCAs, clinical impact curves were plotted to help us more intuitively understand the nomogram models’ significant value. Moreover, all participants were divided by risk scores quartile into low‐, medium‐, and high‐risk subgroups, by which the clinical utility of the nomograms could be measured.

### 2.4 Statistical analysis

All the statistical analyses were calculated in R software (version 3.6.1, https://www.r‐proje ct.org/). The Chi squared tests or Fisher's exact tests were used to compare categorical variables. Survival variables were compared by Wilcoxon tests. The nomograms, C‐index, calibration curves, Kaplan‐Meier curves, cumulative incidence curves DCAs, and clinical impact curves were conducted using R 3.6.1 with relevant packages and functions, such as rms, rmda, survival, cmprsk, and stdca (https://www.mskcc.org/departments/epidemiology‐biostatistics/health‐outcomes/tutorial‐r). A two‐tailed value of *P* < .05 was statistically significant.

## 3 RESULTS

### 3.1 Patients and tumor characteristics

According to the screening criteria, 17 516 patients diagnosed with T1 colorectal cancer who underwent surgical resection during 2004‐2016 from the SEER database, were finally included in this study. There were two study groups, the study cohort N (T1N0‐2M0 stage CRC, n = 17 309) and the study cohort M (T1 CRC, n = 17 516). The median follow‐up time of the study cohort N and M were 54.0 months (interquartile range 24.0‐89.0 months) and 53.0 months (interquartile range 23.0‐89.0 months), respectively. LNM occurred in 2291 of 17 309 patients (13.24%) in the study cohort N and DM was present in 207 of 17 516 patients (1.18%) in the study cohort M. The characteristics of the patients are shown in Table 1.

**Table 1:** Clinicopathological characteristics of patients with T1 colorectal carcinoma

Clinicopathological variables	Nt (%)	Ne (%)	Nne (%)	*P*	Mt (%)	Me (%)	Mne (%)	*P*
	N = 17 309	N = 2291	N = 15 018		N = 17 516	N = 207	N = 17 309	
Year of diagnosis				.035				.006
2004‐2007	3526 (20.37)	505 (22.04)	3021 (20.12)		3586 (20.47)	60 (28.99)	3526 (20.37)	
2008‐2011	5735 (33.13)	772 (33.70)	4963 (33.05)		5802 (33.12)	67 (32.37)	5735 (33.13)	
2012‐2016	8048 (46.50)	1014 (44.26)	7034 (46.84)		8128 (46.40)	80 (38.65)	8048 (46.50)	
Age at diagnosis				<.001				<.001
18‐49	1885 (10.89)	355 (15.50)	1530 (10.19)		1931 (11.02)	46 (22.22)	1885 (10.89)	
50‐64	7149 (41.30)	1083 (47.27)	6066 (40.39)		7238 (41.32)	89 (43.00)	7149 (41.30)	
65‐79	6423 (37.11)	698 (30.47)	5725 (38.12)		6476 (36.97)	53 (25.60)	6423 (37.11)	
80+	1852 (10.70)	155 (6.77)	1697 (11.30)		1871 (10.68)	19 (9.18)	1852 (10.70)	
Race				.012				.816
White	13 729 (79.32)	1775 (77.48)	11 954 (79.60)		13 894 (79.32)	165 (79.71)	13 729 (79.32)	
Black	1953 (11.28)	267 (11.65)	1686 (11.23)		1973 (11.26)	20 (9.66)	1953 (11.28)	
Asian/Pacific Islander	1521 (8.79)	239 (10.43)	1282 (8.53)		1542 (8.80)	21 (10.14)	1521 (8.79)	
American Indian/Alaska Native	106 (0.61)	10 (0.44)	96 (0.64)		107 (0.61)	1 (0.48)	106 (0.61)	
Gender				.009				.641
Female	8686 (50.18)	1208 (52.73)	7478 (49.79)		8786 (50.16)	100 (48.31)	8686 (50.18)	
Male	8623 (49.82)	1083 (47.27)	7540 (50.21)		8730 (49.84)	107 (51.69)	8623 (49.82)	
Marital status				.002				.421
Married	10 484 (60.57)	1460 (63.73)	9024 (60.09)		10 609 (60.57)	125 (60.39)	10 484 (60.57)	
Unmarried	5903 (34.10)	731 (31.91)	5172 (34.44)		5978 (34.13)	75 (36.23)	5903 (34.10)	
Unknown	922 (5.33)	100 (4.36)	822 (5.47)		929 (5.30)	7 (3.38)	922 (5.33)	
Tumor location				<.001				<.001
Right side	6655 (38.45)	668 (29.16)	5987 (39.87)		6708 (38.30)	53 (25.60)	6655 (38.45)	
Left side	9451 (54.60)	1520 (66.35)	7931 (52.81)		9591 (54.76)	140 (67.63)	9451 (54.60)	
Not stated	1203 (6.95)	103 (4.50)	1100 (7.32)		1217 (6.95)	14 (6.76)	1203 (6.95)	
Histology				<.001				<.001
Adenocarcinoma	16 782 (96.96)	2166 (94.54)	14 616 (97.32)		16 971 (96.89)	189 (91.30)	16 782 (96.96)	
Mucinous adenocarcinoma	393 (2.27)	91 (3.97)	302 (2.01)		402 (2.30)	9 (4.35)	393 (2.27)	
Other/Not stated	134 (0.77)	34 (1.48)	100 (0.67)		143 (0.82)	9 (4.35)	134 (0.77)	
Tumor size				<.001				<.001
1‐9 mm	3280 (18.95)	362 (15.80)	2918 (19.43)		3289 (18.78)	9 (4.35)	3280 (18.95)	
10‐19 mm	3797 (21.94)	547 (23.88)	3250 (21.64)		3817 (21.79)	20 (9.66)	3797 (21.94)	
20‐29 mm	2598 (15.01)	356 (15.54)	2242 (14.93)		2617 (9.18)	19 (9.18)	2598 (15.01)	
30 + mm	2810 (16.23)	476 (20.78)	2334 (15.54)		2906 (46.38)	96 (46.38)	2810 (16.23)	
Not stated	4824 (27.87)	550 (24.01)	4274 (28.46)		4887 (30.43)	63 (30.43)	4824 (27.87)	
Regional nodes examined				.02				.063
12‐14	5457 (31.53)	673 (29.38)	4784 (31.86)		5507 (31.44)	50 (24.15)	5457 (31.53)	
15‐19	5858 (33.84)	772 (33.70)	5086 (33.87)		5932 (33.87)	74 (35.75)	5858 (33.84)	
20+	5994 (34.63)	846 (36.93)	5148 (34.28)		6077 (34.69)	83 (40.10)	5994 (34.63)	
N classification				NA				<.001
N0	NA	NA	NA		15 105 (86.24)	87 (42.03)	15 018 (86.76)	
N1	NA	NA	NA		2087 (11.91)	70 (33.82)	2017 (11.65)	
N2	NA	NA	NA		324 (1.85)	50 (24.15)	274 (1.58)	
Grade				<.001				<.001
Well differentiated	3079 (17.79)	239 (10.43)	2840 (18.91)		3091 (17.65)	12 (5.80)	3079 (17.79)	
Moderately differentiated	11 233 (64.90)	1551 (67.70)	9682 (64.47)		11 362 (64.87)	129 (62.32)	11 233 (64.90)	
Poorly differentiated	1229 (7.10)	329 (14.36)	900 (5.99)		1256 (7.17)	27 (13.04)	1229 (7.10)	
Undifferentiated	168 (0.97)	30 (1.31)	138 (0.92)		175 (1.00)	7 (3.38)	168 (0.97)	
Not stated	1600 (9.24)	142 (6.20)	1458 (9.71)		1632 (9.32)	32 (15.46)	1600 (9.24)	
Survival status				<.001				<.001
Alive	14 919 (86.19)	1919 (83.76)	13 000 (86.56)		15 006 (85.67)	87 (42.03)	14 919 (86.19)	
Dead of cancer	633 (3.66)	184 (8.03)	449 (2.99)		739 (4.22)	106 (51.21)	633 (3.66)	
Dead of other cause	1757 (10.15)	188 (8.21)	1569 (10.45)		1771 (10.11)	14 (6.76)	1757 (10.15)	
CEA				<.001				<.001
Positive	1094 (6.32)	192 (8.38)	902 (6.01)		1188 (6.78)	94 (45.41)	1094 (6.32)	
Negative	7229 (41.76)	1079 (47.10)	6150 (40.95)		7271 (41.51)	42 (20.29)	7229 (41.76)	
Borderline/Unknown	8986 (51.92)	1020 (44.52)	7966 (53.04)		9057 (51.71)	71 (34.30)	8986 (51.92)	
Follow‐up time				.754				<.001
	54 (24‐89)	54 (23‐90)	53 (24‐89)		53 (23‐89)	31 (14.5‐58)	54 (24‐89)	

Note

Me(%), number of DM events; Mne(%), number of non‐DM events;Mt(%), total number of the cohort M; Ne(%), number of LNM events; Nne(%), number of non‐LNM events; Nt(%), total number of the cohort N.

Abbreviations: CEA, carcinoembryonic antigen; N, node.

### 3.2 Independent risk factors of lymph nodes metastasis and development of the nomogram

Independent risk factors for LNM were determined by univariable and multivariable binary logistic regression analyses. These significant risk factors for LNM included year of diagnosis, age at diagnosis, race, gender, marital status, tumor location, histology, tumor size, number of regional nodes examined, grade, survival status, and CEA (Table 2). In terms of age, a decreasing LNM risk was detected in older patients, especially for age over 80 years (OR = 0.46, 95%CI = 0.37‐0.57, *P* < .001). Patients with mucinous carcinoma had significantly higher risk of LNM than patients with adenocarcinoma (OR = 2.19, 95%CI = 1.70‐2.80, *P* < .001). Compared with patients who had well‐differentiated CRC, those with poorly differentiated (OR = 3.99, 95%CI = 3.31‐4.81, *P* < .001), and undifferentiated carcinoma (OR = 2.33, 95%CI = 1.50‐3.53, *P* < .001) were at higher risk of LNM.

**Table 2:** Logistic regression analysis of the risk factors for lymph nodes metastasis in T1N0‐2M0 colorectal carcinoma

Clinicopathological variables	Univariate analysis		Multivariate analysis	
	OR (95%CI)	*P*	OR (95%CI)	*P*
Year of diagnosis				
2004‐2007	Reference		Reference	
2008‐2011	0.93 (0.82‐1.05)	.243	0.92 (0.82‐1.05)	.211
2012‐2016	0.86 (0.77‐0.97)	.012	0.85 (0.76‐0.96)	.009
Age at diagnosis				
18‐49	Reference		Reference	
50‐64	0.77 (0.21‐0.26)	<.001	0.86 (0.75‐0.99)	.036
65‐79	0.53 (0.46‐0.60)	<.001	0.61 (0.53‐0.71)	<.001
80+	0.39 (0.32‐0.48)	<.001	0.46 (0.37‐0.57)	<.001
Race				
White	Reference		Reference	
Black	1.07 (0.93‐1.22)	.360	1.11 (0.96‐1.29)	.141
Asian/Pacific Islander	1.26 (1.08‐1.45)	.002	1.19 (1.02‐1.39)	.021
American Indian/Alaska Native	0.70 (0.34‐1.28)	.287	0.79 (0.38‐1.46)	.491
Gender				
Female	Reference		Reference	
Male	0.89 (0.81‐0.97)	.009	0.81 (0.74‐0.89)	<.001
Marital status				
Married	Reference		Reference	
Unmarried	0.87 (0.79‐0.96)	.005	0.90 (0.82‐1.00)	.045
Unknown	0.75 (0.60‐0.93)	.009	0.78 (0.62‐0.97)	.029
Tumor location				
Right side	Reference		Reference	
Left side	1.72 (1.56‐1.89)	<.001	1.59 (1.43‐1.76)	<.001
Not stated	0.84 (0.67‐1.04)	.114	0.86 (0.69‐1.07)	.186
Histology				
Adenocarcinoma	Reference			
Mucinous adenocarcinoma	2.03 (1.59‐2.57)	<.001	2.19 (1.70‐2.80)	<.001
Other/Not stated	2.29 (1.53‐3.36)	<.001	1.92 (1.25‐2.89)	.002
Tumor size				
1‐9 mm	Reference		Reference	
10‐19 mm	1.36 (1.18‐1.56)	<.001	1.24 (1.07‐1.44)	.004
20‐29 mm	1.28 (1.09‐1.50)	.002	1.17 (0.99‐1.37)	.059
30 + mm	1.64 (1.42‐1.91)	<.001	1.56 (1.34‐1.81)	<.001
Not stated	1.04 (0.90‐1.19)	.610	0.95 (0.92‐1.11)	.526
Regional nodes examined				
12‐14	Reference		Reference	
15‐19	1.08 (0.97‐1.21)	.178	1.08 (0.96‐1.20)	.211
20+	1.17 (1.05‐1.30)	.005	1.16 (1.03‐1.30)	.012
Grade				
Well differentiated	Reference		Reference	
Moderately differentiated	1.90 (1.65‐2.20)	<.001	1.76 (1.53‐2.04)	<.001
Poorly differentiated	4.34 (3.62‐5.22)	<.001	3.99 (3.31‐4.81)	<.001
Undifferentiated	2.58 (1.68‐3.86)	<.001	2.33 (1.50‐3.53)	<.001
Not stated	1.16 (0.93‐1.44)	.187	1.14 (0.91‐1.43)	.237
CEA				
Positive	Reference		Reference	
Negative	0.82 (0.70‐0.98)	.025	0.83 (0.70‐0.99)	.033
Borderline/Unknown	0.60 (0.51‐0.71)	<.001	0.65 (0.55‐0.78)	<.001

Abbreviations: 95%CI, 95% confidence intervals; CEA, carcinoembryonic antigen; OR, odd ratio.

**Figure 2:**
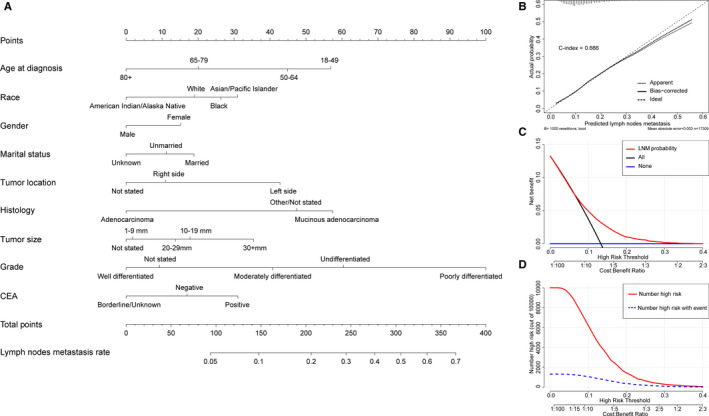
Nomogram, calibration curve, decision curve analysis, and clinical impact curve for predicting LNM in patients with T1N0‐2M0 colorectal carcinoma. There are nine factors in LNM prediction nomogram (A). Calibration curve (B) for predicting LNM is shown and C‐index = 0.666. The diagonal line shows equality between the actual and predicted LNM probability. With the solid line close to the diagonal line, the plot reveals excellent agreement between the probability of nomogram prediction and actual observations. The decision curve (C) of the nomogram for predicting LNM were plotted. The *x*‐axis represents the threshold probability and the *y*‐axis shows the net benefit. The horizontal blue line represents one extreme situation that no patients suffered LNM, and the black line indicates the other extreme situation that all patients experienced LNM. As clinical impact curve (D) shows, the number of high‐risk patients and the number of high‐risk patients with event were plotted by different threshold probability in a population

To more intuitively display the risk factors for LNM in CRCs with T1N0‐2M0 stage, a nomogram model was established (Figure 2A). Additionally, scores assignments and predictive probability for each variable in the nomogram were calculated in Table 3. According to the LNM nomogram, tumor grade accounted for the largest proportion, followed by age, histology, tumor location, tumor size, race, CEA, marital status, and gender. The calibration curve showed an effectively predictive accuracy of the nomogram, with a C‐index of 0.666 (Figure 2B). Moreover, DCA and CIC were performed on the LNM nomogram in the study cohort N (Figure 2C,D), showing that threshold probabilities of 0‐0.3 were the most beneficial for predicting LNM by our nomogram.

**Table 3:** Nomogram score of independent factors for LNM, DM, and OS in T1 colorectal carcinoma

Clinicopathological variables	Nomogram score		
	Lymph nodes metastasis	Distant metastasis	Overall survival
Age at diagnosis			
18‐49	57	27	0
50‐64	45	14	18
65‐79	20	0	56
80+	0	8	100
Race			
White	19		18
Black	26		29
Asian/Pacific Islander	31		0
American Indian/Alaska Native	0		21
Gender			
Female	15		0
Male	0		15
Marital status			
Married	19		1
Unmarried	11		16
Unknown	0		0
Tumor location			
Right side	11		
Left side	43		
Not stated	0		
Histology			
Adenocarcinoma	0		0
Mucinous adenocarcinoma	57		3
Other/Not stated	47		14
Tumor size			
1‐9 mm	2	0	1
10‐19 mm	18	15	5
20‐29 mm	14	20	8
30 + mm	35	68	13
Not stated	0	47	0
N classification			
N0		0	0
N1		57	13
N2		100	29
M classification			
M0			0
M1			68
Grade			
Well differentiated	0	0	0
Moderately differentiated	41	29	2
Poorly differentiated	100	39	0
Undifferentiated	60	57	17
Not stated	9	48	1
CEA			
Positive	31	84	23
Negative	17	0	0
Borderline/Unknown	0	11	8

Abbreviations: CEA, carcinoembryonic antigen; M, metastasis; N, node.

### 3.3 Predictors of distant metastasis and construction of the nomogram

We further evaluated the association between DM and clinicopathological variables. Univariable and multivariable binary logistic regression analyses showed that age at diagnosis, tumor size, N classification, grade, and CEA were significant risk factors for patients with T1 CRC (Table 4). Patients aged 65‐79 had significantly lower risk of DM than patients aged 18‐49 (OR = 0.53, 95%CI = 0.34‐0.83, *P* = .005). Compared with patients whose tumor size were 1‐9mm, those with over 30 mm of tumor size (OR = 6.34, 95%CI = 3.29‐13.77, *P* < .001) were at higher risk of DM. Patients were especially at a higher risk of DM when they suffered from lymph nodes metastasis (N1, OR = 4.77, 95%CI = 3.40‐6.67, *P* < .001; N2, OR = 15.25, 95%CI = 9.91‐23.27, *P* < .001). The DM often occurred when the tumor grade was moderately differentiated (OR = 2.18, 95%CI = 1.22‐4.28, *P* = .014), poorly differentiated (OR = 2.76 95%CI = 1.36‐5.93, *P* = .007), or undifferentiated (OR = 4.37, 95%CI = 1.35‐12.95, *P* < .001). CEA‐negative patients were less prone to DM compared with the CEA‐positive (OR = 0.10, 95%CI = 0.07‐0.14, *P* < .001).

**Table 4:** Logistic regression analysis of the risk factors for distant metastasis in T1 colorectal carcinoma

Clinicopathological variables	Univariate analysis		Multivariate analysis	
	OR (95%CI)	*P*	OR (95%CI)	*P*
Year of diagnosis				
2004‐2007	Reference		Reference	
2008‐2011	0.69 (0.48‐0.98)	.036	0.71 (0.49‐1.05)	.082
2012‐2016	0.58 (0.42‐0.82)	.002	0.75 (0.52‐1.08)	.123
Age at diagnosis				
18‐49	Reference		Reference	
50‐64	0.51 (0.36‐0.74)	<.001	0.74 (0.50‐1.12)	.145
65‐79	0.34 (0.23‐0.51)	<.001	0.53 (0.34‐0.83)	.005
80+	0.42 (0.24‐0.71)	.002	0.67 (0.36‐1.21)	.191
Race				
White	Reference			
Black	0.85 (0.52‐1.32)	.501		
Asian/Pacific Islander	1.15 (0.71‐1.77)	.552		
American Indian/Alaska Native	0.78 (0.04‐3.55)	.81		
Gender				
Female	Reference			
Male	1.08 (0.82‐1.42)	.592		
Marital				
Married	Reference			
Unmarried	1.07 (0.80‐1.42)	.665		
Unknown	0.64 (0.27‐1.27)	.247		
Tumor location				
Right side	Reference		Reference	
Left side	1.86 (1.36‐2.58)	<.001	1.43 (1.00‐2.07)	.054
Not stated	1.46 (0.78‐2.57)	.209	1.73 (0.87‐3.21)	.099
Histology				
Adenocarcinoma	Reference		Reference	
Mucinous adeocarcinoma	2.03 (0.96‐3.77)	.04	1.05 (0.45‐2.18)	.902
Other/Not stated	5.96 (2.78‐11.24)	<.001	1.88 (0.76‐4.17)	.146
Tumor size				
1‐9 mm	Reference		Reference	
10‐19 mm	1.92 (0.90‐4.44)	.105	1.5 (0.69‐3.51)	.323
20‐29 mm	2.67 (1.24‐6.20)	.016	1.7 (0.77‐4.03)	.206
30 + mm	12.45 (6.64‐26.58)	<.001	6.34 (3.29‐13.77)	<.001
Not stated	4.76 (2.49‐10.28)	<.001	3.46 (1.76‐7.63)	<.001
Regional nodes examined				
12‐14	Reference		Reference	
15‐19	1.38 (0.96‐1.99)	.081	1.19 (0.81‐1.75)	.387
20+	1.51 (1.07‐2.16)	.022	1.24 (0.85‐1.82)	.273
N classification				
N0	Reference		Reference	
N1	5.99 (4.35‐8.23)	<.001	4.77 (3.40‐6.67)	<001
N2	31.50 (21.69‐45.33)	<.001	15.25 (9.91‐23.27)	<.001
Grade				
Well differentiated	Reference		Reference	
Moderately differentiated	2.95 (1.70‐5.63)	<001	2.18 (1.22‐4.28)	.014
Poorly differentiated	5.64 (2.91‐11.58)	<.001	2.76 (1.36‐5.93)	.007
Undifferentiated	10.69 (3.93‐26.93)	<.001	4.37 (1.35‐12.95)	.010
Not stated	5.13 (2.71‐10.40)	<.001	3.61 (1.82‐7.64)	<.001
CEA				
Positive	Reference		Reference	
Negative	0.07 (0.05‐0.10)	<.001	0.10 (0.07‐0.14)	<.001
Borderline/Unknown	0.09 (0.07‐0.13)	<.001	0.13 (0.09‐0.19)	<.001

Abbreviations: 95%CI, 95% confidence intervals; CEA, carcinoembryonic antigen; N, node; OR, odd ratio.

**Figure 3:**
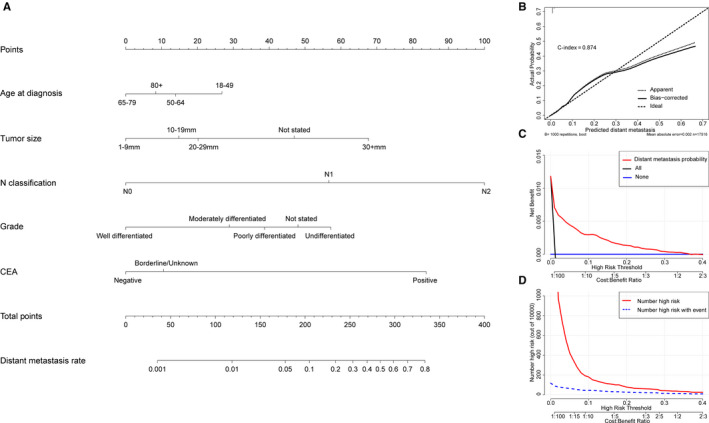
Nomogram, calibration curve, decision curve analysis, and clinical impact curve for predicting DM in patients with T1 colorectal carcinoma. There are five factors in DM prediction nomogram (A). Calibration curve (B) for predicting DM is shown and C‐index = 0.874. The diagonal line shows equality between the actual and predicted DM probability. With the solid line close to the diagonal line, the plot reveals excellent agreement between the probability of nomogram prediction and actual observations. The decision curve (C) of the nomogram for predicting DM were plotted. The *x*‐axis represents the threshold probability and the *y* axis shows the net benefit. The horizontal blue line represents one extreme situation that no patients suffered DM, and the black line indicates the other extreme situation that all patients experienced DM. As clinical impact curve (D) shows, the number of high‐risk patients and the number of high‐risk patients with event were plotted by different threshold probability in a population

Based on the significant risk factors identified in the multivariable regression analysis, a nomogram was constructed to predict the probability of DM in patients with T1 colorectal carcinoma (Figure 3A). Each variable was assigned a score and the estimated DM possibility were calculated by the total scores in Table 3. N classification made the largest contribution in the DM nomogram, followed by CEA, tumor size, grade, and age at diagnosis. The calibration plot showed a relative satisfactory predictive accuracy of the nomogram (Figure 3B). The nomogram displayed a C‐index of 0.874, which effectively predicted the risk of DM from T1 CRC. Furthermore, we found that threshold probabilities of 0‐0.3 were the most beneficial for predicting DM by DCA and CIC plotted on the DM nomogram in the study cohort M (Figure 3C,D).

### 3.4 Survival analyses based on the Kaplan‐Meier and gray method

**Figure 4:**
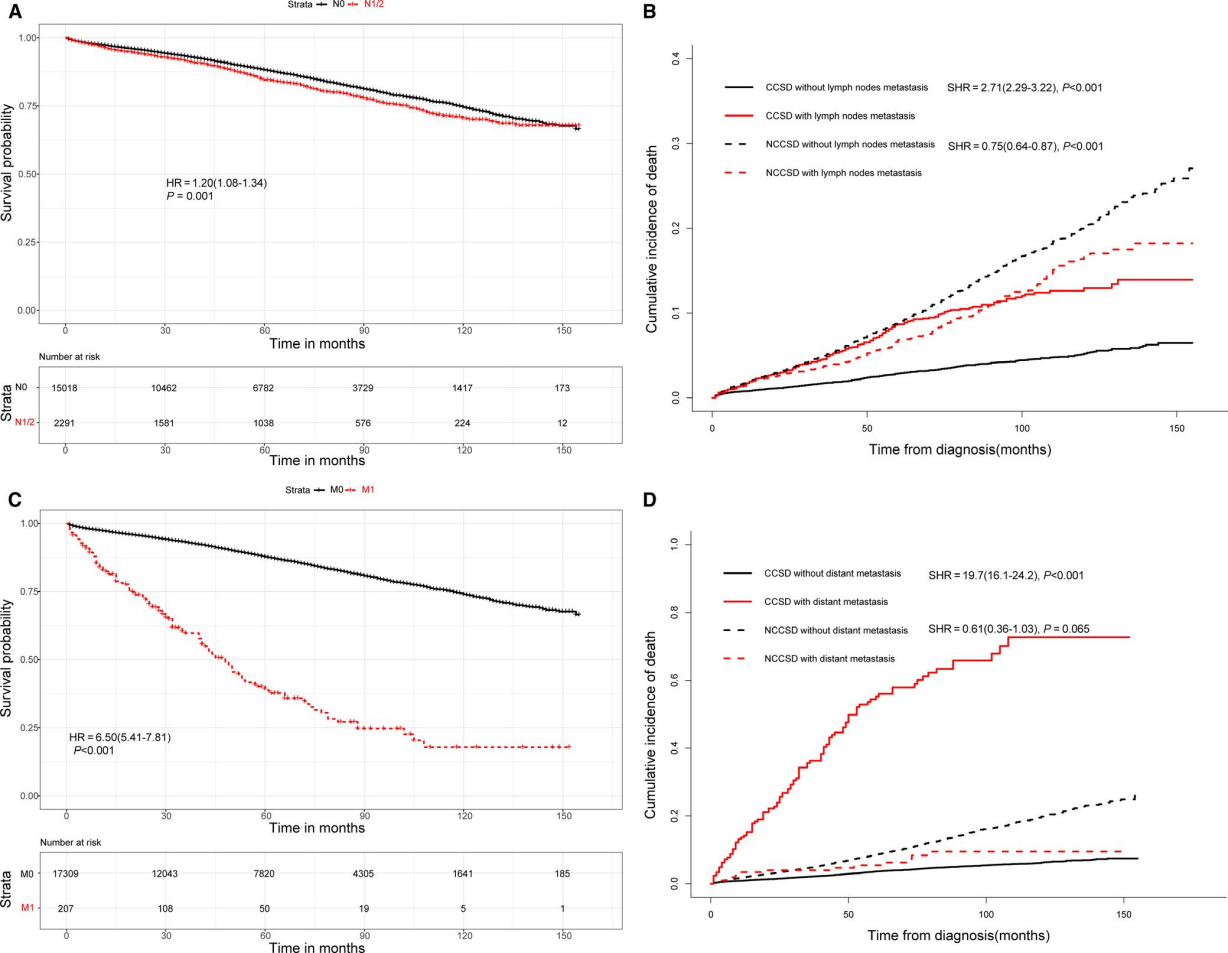
Effect of lymph nodes metastasis on overall survival (A) and cancer‐specific survival (B) in T1 colorectal cancer. Impact of distant metastasis on overall survival (C) and cancer‐specific survival (D) in T1 colorectal cancer

The Kaplan‐Meier and Gray method were used to determine the impact of lymph nodes metastasis and distant metastasis on the survival. Kaplan‐Meier curves showed that positive lymph node involvement (hazard ratio (HR) = 1.20, 95%CI = (1.08‐1.34), *P* = .001) and distant metastasis (HR = 6.50, 95%CI = (5.41‐7.81), *P* < .001) were significantly associated with overall survival (Figure 4A,C). Consistently, we found that LNM (subdistribution hazard ratio (SHR) = 2.71, 95%CI=(2.29‐3.22), *P* < .001) and DM (SHR = 19.7, 95%CI = (16.1‐24.2), *P* < .001) were significantly connected with cancer‐specific death using Gray method (Figure 4B,D).

### 3.5 Prognostic factors for T1 colorectal cancer and establishment of the nomogram

Using univariable and multivariable COX regression analyses, we found that age at diagnosis, race, gender, marital status, histology, tumor size, number of regional nodes examined, N classification, M classification, grade, and CEA were significant prognostic factors for overall survival in T1 colorectal cancer (Table 5). Compared with patients aged 18‐49, those aged 50‐64 (HR = 1.58, 95%CI = 1.28‐1.96, *P* < .001), aged 65‐79 (HR = 4.18, 95%CI = 3.40‐5.14, *P* < .001), and aged over 80 (HR = 12.97, 95%CI = 10.47‐16.05, *P* < .001) were at higher death risk. The death probability often increased when lymph nodes metastasis occurred (N1, HR = 1.41, 95%CI = 1.25‐1.58, *P* < .001; N2, HR = 2.12, 95%CI = 1.72‐2.60, *P* < .001). Patients with distant metastasis had significantly higher death risk than patients without distant metastasis (HR = 5.82, 95%CI = 4.75‐7.11, *P* < .001).

**Table 5:** COX regression analysis of the prognostic factors for overall survival in T1 colorectal carcinoma

Clinicopathological variables	Univariate analysis		Multivariate analysis	
	HR (95%CI)	*P*	HR (95%CI)	*P*
Year of diagnosis				
2004‐2007	Reference		Reference	
2008‐2011	0.96 (0.88‐1.06)	.446	1.02 (0.93‐1.12)	.609
2012‐2016	0.80 (0.70‐0.91)	<.001	0.89 (0.78‐1.01)	.080
Age at diagnosis				
18‐49	Reference		Reference	
50‐64	1.48 (1.12‐1.84)	<.001	1.58 (1.28‐1.96)	<.001
65‐79	3.76 (3.06‐4.61)	<.001	4.18 (3.40‐5.14)	<.001
80+	11.81 (9.60‐14.53)	<.001	12.97 (10.47‐16.05)	<.001
Race				
White	Reference		Reference	
Black	1.08 (0.96‐1.22)	.187	1.32 (1.17‐1.49)	<.001
Asian/Pacific Islander	0.57 (0.48‐0.69)	<.001	0.62 (0.52‐0.75)	<.001
American Indian/Alaska Native	0.94 (0.55‐1.62)	.825	1.05 (0.61‐1.82)	.859
Gender				
Female	Reference		Reference	
Male	1.08 (1.00‐1.17)	.046	1.45 (1.34‐1.58)	<.001
Marital				
Married	Reference		Reference	
Unmarried	1.79 (1.65‐1.94)	<.001	1.45 (1.33‐1.57)	<.001
Unknown	1.07 (0.88‐1.32)	.496	0.97 (0.79‐1.19)	.786
Tumor location				
Right side	Reference		Reference	
Left side	0.67 (0.61‐0.72)	<.001	0.98 (0.90‐1.07)	.608
Not stated	0.81 (0.69‐0.95)	.012	0.94 (0.80‐1.11)	.457
Histology				
Adenocarcinoma	Reference		Reference	
Mucinous adenocarcinoma	1.46 (1.18‐1.79)	<.001	1.07 (0.86‐1.32)	.542
Other/Not stated	1.81 (1.30‐2.53)	<.001	1.45 (1.03‐2.05)	.036
Tumor size				
1‐9 mm	Reference		Reference	
10‐19 mm	1.24 (1.08‐1.42)	.002	1.12 (0.98‐1.29)	.107
20‐29 mm	1.39 (1.21‐1.61)	<.001	1.2 (1.04‐1.39)	012
30 + mm	1.79 (1.57‐2.05)	<.001	1.36 (1.19‐1.56)	<.001
Not stated	0.94 (0.82‐1.08)	.372	0.97 (0.84‐1.11)	.638
Regional nodes examined				
12‐14	Reference		Reference	
15‐19	0.89 (0.81‐10.98)	.02	0.89 (0.81‐0.98)	.016
20+	0.86 (0.78‐0.96)	.002	0.88 (0.79‐0.97)	.008
N classification				
N0	Reference		Reference	
N1	1.21 (1.08‐1.36)	.001	1.41 (1.25‐1.58)	<.001
N2	2.55 (2.10‐3.08)	<.001	2.12 (1.72‐2.60)	<.001
M classification				
M0	Reference		Reference	
M1	6.50 (5.41‐7.81)	<001	5.82 (4.75‐7.11)	<.001
Grade				
Well differentiated	Reference		Reference	
Moderately differentiated	1.04 (0.94‐1.16)	.462	1.04 (0.93‐1.15)	.500
Poorly differentiated	1.14 (0.97‐1.34)	.119	0.99 (0.84‐1.17)	.907
Undifferentiated	1.88 (1.33‐2.66)	<.001	1.57 (1.10‐2.25)	.013
Not stated	0.89 (0.75‐1.04)	14	1.01 (0.85‐1.19)	916
CEA				
Positive	Reference		Reference	
Negative	0.38 (0.34‐0.44)	<.001	0.55 (0.48‐0.64)	<.001
Borderline/Unknown	0.49 (0.43‐0.56)	<.001	0.67 (0.59‐0.77)	<.001

Abbreviations: 95%CI, 95% confidence intervals; CEA, carcinoembryonic antigen; HR, hazard ratio; M, metastasis; N, node.

To study the colorectal cancer‐specific death (CCSD) of T1 colorectal carcinoma, competing risk model was performed. These significant prognostic factors included age at diagnosis, race, marital status, tumor size, N classification, M classification, and CEA (Table 6). In terms of age, an increasing CCSD risk was detected in older patients, especially for age 65‐79 years (SHR = 1.79, 95%CI = 1.39‐2.31, *P* < .001) and age over 80 years (SHR = 3.23, 95%CI = 2.43‐4.30, *P* < .001). Patients of black descent were more prone to CCSD compared with Caucasians (SHR = 1.70, 95%CI = 1.39‐2.08, *P* < .001). Unmarried status was a significant factor associated with CCSD (SHR = 1.18, 95%CI = 1.01‐1.39, *P* = .036). Compared with patients whose tumor size were 1‐9mm, those with over 30mm (SHR = 1.62, 95%CI = 1.24‐2.11, *P* < .001) were at higher risk of CCSD. Patients of higher N stages suffered from a higher risk of CCSD (N1, SHR = 2.18, 95%CI = 1.80‐2.63, *P* < .001; N2, SHR = 4.45, 95%CI = 3.36‐5.89, *P* < .001). The CCSD often occurred when the CRC tumor existed distant metastasis (SHR = 9.12, 95%CI = 6.96‐11.96, *P* < .001). Patients with lower CEA levels were at lower CCSD risk (SHR = 0.57, 95%CI = 0.44‐0.72, *P* < .001).

**Table 6:** Competing risk regression analysis of the prognostic factors for cancer‐specific survival in T1 colorectal carcinoma

	Univariate analysis		Multivariate analysis	
	HR (95%CI)	*P*	HR (95%CI)	*P*
Year of diagnosis				
2004‐2007	Reference		Reference	
2008‐2011	0.76 (0.64‐0.89)	<.001	0.82 (0.69‐0.96)	.016
2012‐2016	0.56 (0.45‐0.70)	<.001	0.61 (0.49‐0.77)	<.001
Age at diagnosis				
18‐49	Reference		Reference	
50‐64	0.91 (0.70‐1.19)	.5	1.16 (0.9‐1.5)	.24
65‐79	1.25 (0.97‐1.62)	.09	1.79 (1.39‐2.31)	<.001
80+	2.23 (1.69‐2.95)	<.001	3.23 (2.43‐4.30)	<.001
Race				
White	Reference		Reference	
Black	1.48 (1.21‐1.81)	<.001	1.70 (1.39‐2.08)	<.001
Asian/Pacific Islander	0.77 (0.57‐1.05)	.095	0.81 (0.6‐1.09)	.17
American Indian/Alaska Native	1.58 (0.71‐3.54)	.26	1.81 (0.9‐3.61)	.094
Gender				
Female	Reference			
Male	1.14 (0.99‐1.31)	.08		
Marital status				
Married	Reference		Reference	
Unmarried	1.40 (1.21‐1.63)	<.001	1.18 (1.01‐1.39)	.036
Unknown	1.06 (0.75‐1.51)	.73	1.05 (0.74‐1.48)	.8
Tumor location				
Right side	Reference			
Left side	1.05 (0.90‐1.22)	.55		
Not stated	0.83 (0.60‐1.15)	.27		
Histology				
Adenocarcinoma	Reference		Reference	
Mucinous adenocarcinoma	1.79 (1.26‐2.56)	.001	1.21 (0.83‐1.76)	.32
Other/Not stated	4.25 (2.76‐6.55)	<.001	2.58 (1.64‐4.05)	<.001
Tumor size				
1‐9 mm	Reference		Reference	
10‐19 mm	1.33 (1.01‐1.74)	.043	1.13 (0.86‐1.49)	.37
20‐29 mm	1.55 (1.16‐2.06)	.003	1.25 (0.94‐1.66)	.13
30 + mm	2.72 (2.11‐3.50)	<001	1.62 (1.24‐2.11)	<.001
Not stated	1.19 (0.92‐1.54)	.2	1.06 (0.81‐1.38)	.67
Regional nodes examined				
12‐14	Reference			
15‐19	0.99 (0.83‐1.19)	.92		
20+	1.05 (0.88‐1.25)	.63		
N classification				
N0	Reference		Reference	
N1	2.53 (2.13‐3.02)	<.001	2.18 (1.80‐2.63)	<.001
N2	8.79 (7.00‐11.05)	<.001	4.45 (3.36‐5.89)	<.001
M classification				
M0	Reference		Reference	
M1	19.7 (5.16.1‐24.2)	<.001	9.12 (6.96‐11.96)	<.001
Grade				
Well differentiated	Reference		Reference	
Moderately differentiated	1.35 (1.09‐1.67)	.007	1.19 (0.96‐1.47)	.12
Poorly differentiated	2.18 (1.63‐2.90)	<.001	1.39 (1.03‐1.89)	.031
Undifferentiated	3.19 (1.80‐5.66)	<.001	1.61 (0.82‐3.17)	.17
Not stated	1.11 (0.81‐1.53)	.52	0.98 (0.71‐1.36)	.92
CEA				
Positive	Reference		Reference	
Negative	0.28 (0.23‐0.35)	<.001	0.57 (0.44‐0.72)	<.001
Borderline/Unknown	0.33 (0.27‐0.40)	<.001	0.64 (0.51‐0.81)	<.001

Abbreviations: 95%CI, 95% confidence intervals; CEA, carcinoembryonic antigen; HR, hazard ratio; M, metastasis; N, node.

**Figure 5:**
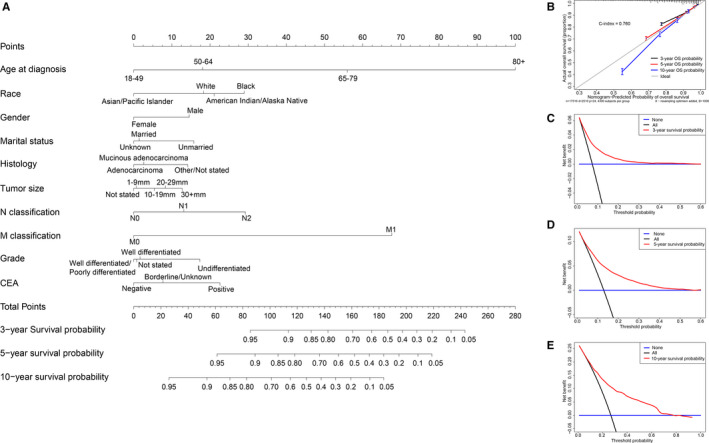
Nomogram, calibration curve, and decision curve analysis for predicting overall survival in patients with T1 colorectal carcinoma. There are ten factors in OS prediction nomogram (A). Calibration curve(B) for predicting 3‐, 5‐ and 10‐year OS is shown and C‐index = 0.760. The diagonal line shows equality between the actual and predicted OS probability. With the solid line close to the diagonal line, the plot reveals excellent agreement between the probability of nomogram prediction and actual observations. The decision curve (C‐E) of the nomogram for predicting 3‐, 5‐ and 10‐year OS were plotted. The *x*‐axis represents the threshold probability and the *y*‐axis shows the net benefit. The horizontal blue line represents one extreme situation that all patients were alive, and the black line indicates the other extreme situation that all patients were dead

The significant factors identified by the COX regression analyses were used to develop the nomogram to predict the probability of overall survival in patients with T1 CRC. The plot of the OS nomogram is shown in Figure 5A. The C‐index of the OS nomogram was 0.760 and the calibration curves revealed relatively excellent agreement between the nomogram prediction and the actual observation for the 3‐, 5‐, and 10‐year OS probability (Figure 5B). Furthermore, using DCA, we found that the most beneficial threshold probabilities for predicting the 3‐, 5‐, and 10‐year death probability were 0‐0.3, 0‐0.5, and 0‐0.7, respectively (Figure 5C,E).

### 3.6 Clinical effects of the risk score in the nomograms

**Figure 6:**
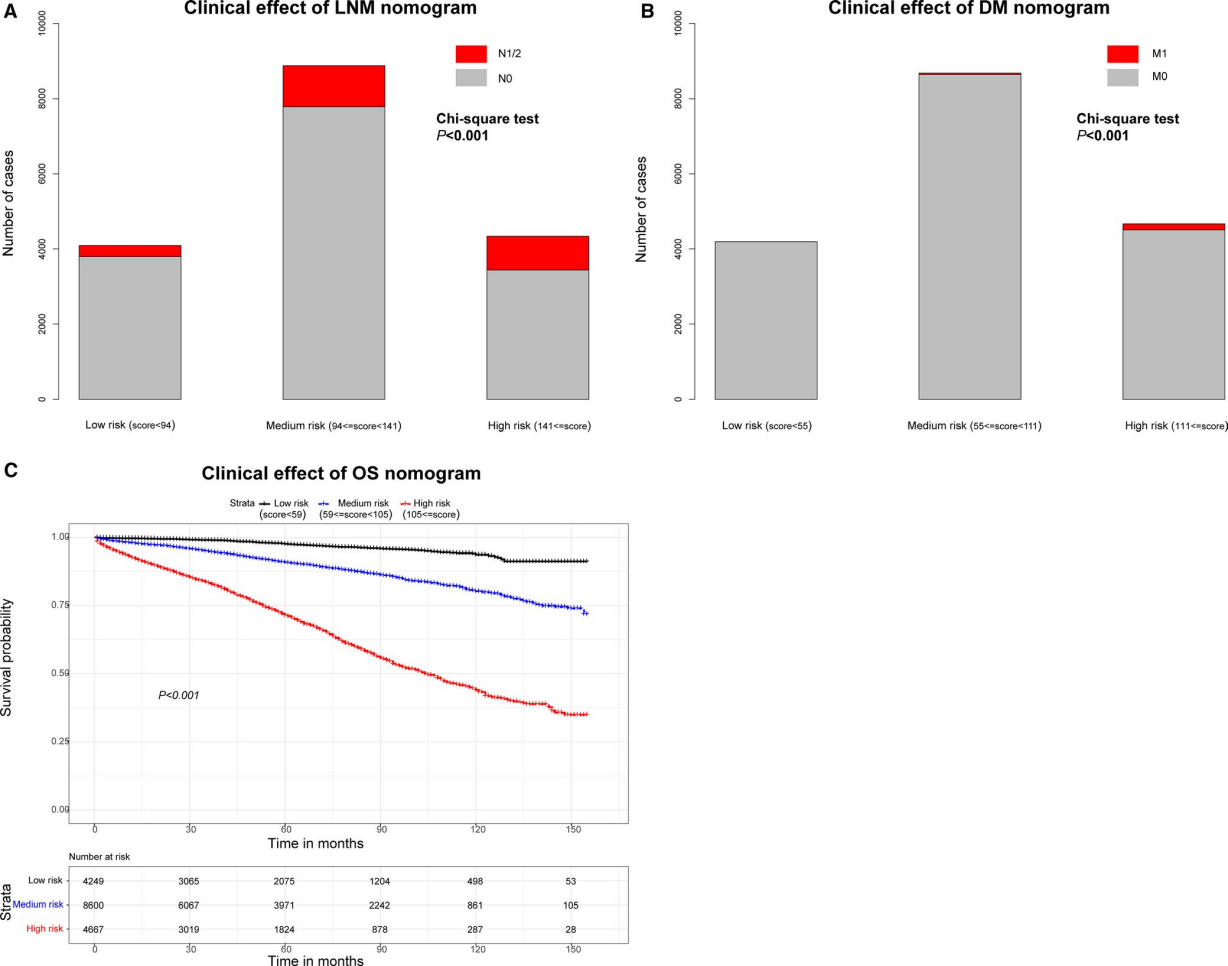
Clinical effects of the risk score in the nomograms. Based on the quartile of risk score, three nomograms divide participants into low‐, medium‐ and high‐ risk subgroup, respectively. Clinical utility of these subgroups for predicting LNM and DM is present by constituent ratio, as well as shows significant difference (A‐B). In term of overall survival, Kaplan‐Meier method is used to found out the significance among the different risk subgroups (C)

Using the 25th and 75th percentile values of the risk score, we classified the cohort N as three subgroups (low risk, score 0‐94; medium risk, 94‐141; high risk, 141‐240). Incidence of LNM among these subgroups was significantly different (*P* < .001) (Figure 6A). Similarly, the cohort M was divided into subgroups as follows: low‐risk DM subgroup, scoring: 0‐55; medium‐risk DM subgroup, scoring: 55‐111; and high‐risk DM subgroup, scoring:111‐336, which significantly discriminated the occurrence of DM (*P* < .001) (Figure 6B). Low‐, medium‐, and high‐ risk subgroup, separated by the score 0‐59, 59‐105, and 105‐272, respectively, showed the statistical significance in overall survival probability (*P* < .001) (Figure 6C).

## 4 DISCUSSION

Due to different clinical features, especially for lymph nodes and metastasis status, patients with submucosal invasive(T1) colorectal cancer have a different prognosis. The treatments are distinct in various situations. About 90% patients with T1 CRC are at stage I and only need to remove the lesions under endoscopy to have a similar prognosis of surgery, avoiding the adverse effects of surgery, and improving the quality of life. T1 CRCs with lymph nodes metastasis, accounting for 6.8%‐17.8%,^7^, ^8^ should undergo surgical resection of primary tumor site and metastatic lymph nodes, as well as accept subsequent adjuvant chemotherapy. However, T1 CRCs with IV stage usually lose the chance of surgery for cure and rely on systemic treatment, including chemotherapy, targeted drugs and immunity therapy. In summary, it is important to distinguish the status of lymph nodes and distant metastasis in clinic. Of course, It is also significant to be able to evaluate overall survival based on clinical pathological characteristics.

Recently such studies are increasing, but there are still many shortcomings and limitations. First, former studies^16^, ^17^ constructed models based on logistic regression analyses and COX regression analyses, but these models could not obtain the prediction probability, making it difficult to apply clinically. Nomogram, as a new form of display, could intuitively predict LNM, DM, and OS. This method forms nomogram diagrams to predict related probabilities, and makes references for further examination and clinical decision making. Second, in the nomogram studies of the T1 CRC population,^18^, ^19^ there are still a lack of integrity. Only the occurrence of LNM or DM is studied, but the prognosis of this population is rarely predicted, which cannot fully reflect the LNM, DM, and OS of T1 CRC patients in one dataset. Third, there are differences in patient inclusion criteria. For example, some studies directly use the data of all T1 CRC patients to assess whether there is lymph node metastasis.^18^, ^19^, ^20^ Although there is a prediction result of LNM, it is of little significance. This is because only nonmetastatic CRC patients have a clinical significance in predicting LNM and prediction of this population will influence treatment decisions. Moreover, these studies^18^, ^19^, ^20^ did not request the number of lymph node biopsies, and usually 12 or more lymph nodes need to be examined to determine the status of the lymph nodes.^12^

Therefore, we divided the included population into N subgroup (T1M0NX CRC for LNM) and M subgroup (T1NXMX CRC for DM and OS). The incidence of LNM and DM, and OS of T1 CRC were analyzed, and the corresponding nomograms were constructed. Three nomograms were established and validated for predicting LNM, DM, and OS in patients with T1 CRC. LNM nomogram includes nine factors: age at diagnosis, race, gender, marital status, tumor location, histology, tumor size, grade, and CEA, whereas DM nomogram incorporates five factors, namely, age at diagnosis, tumor size, N classification, grade, and CEA. OS nomogram for predicting 3‐, 5‐, and 10‐year overall survivals involves 10 factors: age at diagnosis, race, gender, marital status, histology, tumor size, N classification, M classification, grade, and CEA.

All the nomograms indicated good agreement between predictions and observations. C‐index of the LNM nomogram, DM nomogram and OS nomogram were calculated with values of 0.666, 0.874, and 0.760, respectively. These nomograms reveal good clinical utility in the proper threshold probability range. Furthermore, based on the interquartile scores from the nomograms, low‐, medium‐, and high‐risk groups were identified to plot stacked bar charts and Kaplan‐Meier survival curves, which intuitively indicated the discrimination ability of the nomograms.

In this population‐based study, we found that tumor grade III‐IV, mucinous adenocarcinoma, and age 18‐ to 49‐year old accounted for the largest proportion among the LNM nomogram scores. As a significant factor, that degree of differentiation has been reported to be closely associated with LNM in T1 CRC.^9^ In this study, compared with well‐differentiated carcinoma, the LNM risk of poor‐differentiated and undifferentiated cancer rose to approximately 3.99 and 2.33, respectively (both *P* < .001). Consistent with previous findings in T1 CRC,^21^ in this study, patients with mucinous adenocarcinoma increased LNM risk by more than 1 times, in comparison with adenocarcinoma patients. More and more studies^22^, ^23^ reveal that young age at diagnosis is related to an increased risk of LNM in patients with early Colon Cancer. Like these studies, we found that the LNM risk of youngest T1 CRC group (patients age 18‐49 years old) was higher than older patients.

For the DM nomogram, the largest proportion in risk scores were N2 stage, positive CEA, and tumor size over 30mm. Not surprisingly, N classification was a significant predictor for the risk of LNM in T1 CRC. Of note, patients with the worse N stage are more prone to occur distant metastasis. Preoperative CEA has been found to be predictive of distant metastasis in T1 colorectal cancer after radical surgery.^24^ Here, we reveal similar observations, which indicate that cancer with positive CEA prior to treatment is a significant predictive factor for the risk of DM in T1 CRC. Unlike these studies^24^, ^25^ concerning T1 CRC, we found that tumor size was significantly associated with the risk of DM.

In term of the OS nomogram, age over 80 years old, M1 stage and N2 classification take up the largest percentage of the risk score for overall survival. It is not surprising that patients with distant metastasis and N2 classification have poor prognosis. Consistent with our researches, it was reported that older patients with early stage CRC are significantly related to shorter overall survival.^26^, ^27^ Furthermore, we found that LNM in T1 CRC was associated with cancer‐specific death and noncancer‐specific death, whereas DM was only linked with cancer‐specific death.

In this database‐based study, we screen 17 516 eligible patients with a median follow‐up of 53 months from real‐world data. We analyzed the data by appropriate statistical methods and found these convincing conclusions. However, there were some limitations in our study. This was a population‐based retrospective analysis lacking important treatment information, such as surgical methods and chemoradiotherapy protocols. In addition, these data lacked the description of the distant metastasis site and the detection of key molecules of colorectal cancer, like KRAS and BRAF. Finally, these models were developed from the SEER database and were not verified by external data, being continuously modified based on the application in the future.

In conclusion, based on independent risk factors from a large population database, we constructed three nomograms which can accurately predict the LNM, DM, and OS of T1 CRC patients at different stages. Moreover, our nomograms were demonstrated to have high accuracy and reliability by the validation of discrimination and calibration, as well as perform well in clinical utility. Therefore, they can help doctors to make clinical decisions for patients with T1 CRC, including diagnostic investigations, individual treatment, and follow‐up management strategies.

## AUTHORS CONTRIBUTION

KG conceived and designed the study, performed the study, analyzed the data, prepared figures and/or tables, and authored or reviewed drafts of the paper. YF and MS conceived and designed the study, performed the study, analyzed the data. LY, H S. W, and LS performed the study and authored or reviewed drafts of the paper. SR conceived and designed the study, performed the study, authored or reviewed drafts of the paper, and approved the final draft.

## Data Availability

The datasets generated for this study are available in the SEER database (https://seer.cancer.gov/about/overview.html).
